# Thermal, Mechanical, Morphological and Aesthetical Properties of Rotational Molding PE/Pine Wood Sawdust Composites

**DOI:** 10.3390/polym14010193

**Published:** 2022-01-04

**Authors:** Carla I. Martins, Vitória Gil, Sara Rocha

**Affiliations:** Institute for Polymers and Composites, University of Minho, Campus de Azurém, 4800-058 Guimaraes, Portugal; vitoriagilpc@gmail.com (V.G.); sararocha0@gmail.com (S.R.)

**Keywords:** rotational molding, wood-polymer-composites, wood sawdust, polyethylene, thermal properties, morphology, mechanical properties

## Abstract

This research addresses the importance of pine wood sawdust granulometry on the processing of medium-density polyethylene (MDPE)/wood composites by rotational molding and its effects on the morphological, mechanical and aesthetical properties of parts, aiming to contribute for the development of sustainable wood polymer composites (WPC) for rotational molding applications. Pine wood sawdust was sieved (<150, 150, 300, 500, 710, >1000 µm) and analyzed for its physical, morphological and thermal characteristics. Rotational molded parts were produced with matrix/wood ratios from 90/10 to 70/30 wt% considering different wood granulometries. As a natural material, wood changed its color during processing. Granulometries below 500 µm presented better sintering, homogeneity and less part defects. Furthermore, 300–500 µm favored the impact resistance (1316 N), as irregular brick-shaped wood was able to anchor to PE despite the weak interfacial adhesion observed. The increase of wood content from 10 to 30% reduced the impact properties by 40%, as a result of a highly porous structure formed, revealing sintering difficulties during processing. WPC parts of differentiated aesthetics and functionalities were achieved by rotational molding. A clear relationship between wood granulometry and WPC processing, structure and properties was identified.

## 1. Introduction

The human awareness to reduce environmental impact and to reach sustainability, is leading to the effort of industrial waste reuse and the development of ecologically viable materials, from renewable resources. This demand has been growing over the last decades proving that bio economy and circular economy strategies are the solution that can match the scale of an emerging area of polymer composites and the production of high value bio-based products [1,2,3,4]. However, products structural design may be necessary to preserve product functionality, material properties and economic value for longer time [5].

Natural fibers are an example of renewable material that have exceptional attributes such as low density, low cost, abundance in nature, renewability and marketing appeal [6]. They prove to be good alternatives to inorganic materials and a successful solution for wood polymer composites (WPC) that makes use of organic materials with virgin, recycled or green plastics, reducing dependency on petroleum-based raw materials [3,7,8,9]. Moreover, they offer the advantages of enhancing acoustic performance, reduced weight, lower production costs and biodegradability [10]. WPC can be easily processed through typical processing techniques, such as extrusion, injection molding, compression molding or thermoforming and they can also be manufactured like common plastics [11]. More recently, there have been several studies on new production technologies like additive manufacturing based on extrusion processes and laser sintering [12]. WPC can be applied in a wide range of applications but mostly they are found in the construction sectors [13,14,15,16] as lumbers, automotive and electrical sectors, offering cost-competitive products of lighter environmental footprint and unique aesthetic appeal.

The wood components in WPC are generally used in sawdust form or small fibers (sawdust, off-cuts and shavings) as they are a by-product of the wood/timber industry produced by the cutting, sawing or grinding of timber. For that reason, they are irregular in shape and their size distribution can range from Over Size (OS > 710 μm), Coarse Particle Size (CPS = 350~710 μm) and Fine Particle Size (FPS = 177~250 μm), which affects the particles density, porosity and water retention. These physical properties of sawdust are dependent of the wood species [17].

The market of WPC is promising for obtaining rotomolded parts with less polymeric material and aesthetic appearance of natural wood, which can be used in furniture, playgrounds and automotive parts, among others [18,19,20]. Rotational molding is a technique used to produce hollow seamless parts, in which the processing cycle consists in four stages: charging the powder material, heating the material so that polymer can adhere to the mold surfaces and, cooling the part for solidification and demolding. During these stages the mold rotates bi-axially so that material can reach all the surfaces of the mold [21,22,23]. This technique poses a challenge for several materials, including natural ones, due to the possibility of thermo-oxidative degradation during processing, as a result of high temperature and long cycle time applied with impact on aesthetics, morphological and mechanical properties [24,25,26,27,28]. In case of natural lignocellulosic materials, they are formed by a chemical complex of cellulose, hemicellulose, lignin and extractives and inorganics [29,30], which are known to present different degradation profiles depending on its composition and temperature applied. Therefore, molding parameters must be carefully selected to avoid wood thermal degradation. Furthermore, the poor adhesion between polymers and natural fibers and the reduction of the sintering capability of the composite materials, that increases part porosity, are responsible for low mechanical properties [31,32]. Some strategies such as chemical treatment of the fibers and the use of a coupling agent were often used to improve the adhesion of the interface between the polymer matrix and the lignocellulosic filler [9,33,34,35,36].

In case of no treatment for natural and ecological featuring, very recent study from Arribasplata-Seguin et al. [32] shows the need for careful selection of processing parameters. Only with that the sintering process of wood plastic composites is improved with determinant effects on the mechanical properties. There is also an understanding that acceptable mechanical properties can be obtained when low contents of natural materials are incorporated (10–15%) [37,38]. For high fiber contents, fiber wettability by the polymer is difficult and fiber agglomeration may occur. The wood granulometry is also discussed by few authors, indicating better composite performance for small particle sizes, namely maple wood fiber size of 125–250 µm [39] and pine particles between 297–500 μm [32].

Several studies are found in literature dedicated to rotational molding using lignocellulosic materials [19]. Some of these used the following organic materials: jute, sisal and cabuya [31,40,41], wood fiber [32,40], flax [18,41], banana and abaca [42], agave [25,33,35,43], pine wood [32,33], coir [33], maple wood [18,37,38,39,44], hemp [18,34], cork [45] and bamboo [46]. Despite the variety of studies on WPC for rotational molding, there are still few real successful applications using these materials. The lack of studies using pine wood for rotational molding applications and that being a major resource in Portugal motivated the work carried in this study.

Polymer wood composites are a trend and a society demand nowadays. To create differentiated products with high value, ecological, sustainable, aesthetic and functional characteristics there is the need for a complete understanding of the materials used, its processability and the final characteristics of the products obtained. Materials performance depends on wood type, granulometry and content, sintering process and interaction with polymer matrix. This study addresses those subjects focusing on the granulometry and content of pine wood sawdust/MDPE composites produced by rotational molding. The work initiates with the characterization of pine wood sawdust, the production of polymer/wood composites and finally a full characterization of products, from its morphological and mechanical performance to aesthetics, namely, color, visual appearance and defects. This study is in line with other recently published articles in the finding of new materials for rotational molding [9,25,32,45,46].

## 2. Materials and Methods

### 2.1. Pine Wood Sawdust and Polymer Materials

**Figure 1:**
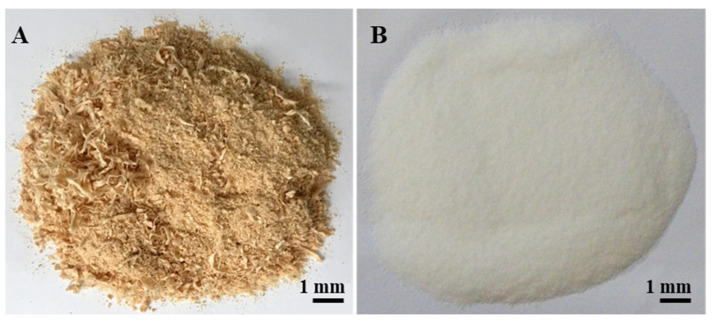
Raw materials: (**A**) Pine wood sawdust and (**B**) MDPE.

Sawdust obtained from wood processing stages of Portuguese pine (Pinus Pinaster) was kindly supplied by A. F. Fábrica de Madeiras, Lda (Braga, Portugal). As received material (Figure 1A) was sieved using a Fritsch Analysette 3 Spartan (Fritsch GmbH, Idar-Oberstein, Germany) sieve to be separated into different granulometries, namely >1000 µm, 710 µm, 500 µm, 300 µm and <150 µm. Prior to the procedure the wood sawdust was dried in a Binder oven (BINDER GmbH, Tuttlingen, Germany) at 50 °C during 6 h. The polymer used in the preparation of the wood-based composites was medium-density polyethylene (MDPE), Advancene EM-3405-UVH, from ETHYDCO (Alexandria, Egypt) with MFI of 5 g/10 min (190 °C/2.16 kg), bulk density of 330–390 kg/m^3^ and maximum particle size of 500 µm (Figure 1B).

### 2.2. Apparent Density

The apparent density of wood sawdust with different granulometries were determined considering the following procedure, following an adaptation of ASTM D1895-96 (2010), method C: 10 g of wood particles were loosely dropped to a 250 mL dry graduated cylindrical cup. The wood sawdust was leveled without compressing and the apparent volume was read. Equation (1) was used to calculate the apparent density in (g/cm^3^), where *W* is the weight of the material in the cylinder (g) and *V* the volume occupied by the material in the measuring cylinder (cm^3^).

(1) $$\mathrm{Apparent}\;\mathrm{density}=\frac{W}{V}$$

### 2.3. Thermal Properties

The thermal properties of wood sawdust in its different granulometries were evaluated by thermogravimetric analysis (TGA) using Pyris 1 thermogravimetric analyzer from Perkin Elmer (Waltham, MA, USA). Experiments were performed in ceramic pans, at a heating rate of 10 °C/min from 30 °C to 800 °C under nitrogen and artificial air atmospheres with a flow rate of 60 mL/min.

### 2.4. Heat Treatment and Color Determination

Wood sawdust of different granulometries were exposed to a heat treatment to evaluate its color change when exposed to high temperatures for a period of time. The temperature and time of exposure were selected according to the typical processing cycle of PE. Therefore, the samples were subjected to temperatures ranging from 170 °C to 230 °C, with 10 °C between them, in a Binder oven for 20, 30 and 40 min, respectively. Upon heat treatment the color was measured using a Spetro-guide BYK portable colorimeter (BYK Geretsried, Germany). The color parameters *L*, *a* and *b* were determined according to the CIELab method. The system is based on the measurement of three coordinates: lightness L* between 0 (black) and 100 (white), a* representing red-green levels (+60 red, −60 green) and b* representing yellow-blue levels (+60 yellow, −60 blue). The color of a material is a mixture of these three parameters. The color variation was calculated according to Equation (2) [47], where ∆*L*, ∆*a* and ∆*b* correspond, to the variation of the coordinates L*, a* and b* relative to the reference samples. The reference sample was considered to be the wood sawdust after separation by granulometries.

(2) $$\Delta E=\sqrt{\Delta L^{2}+\Delta a^{2}+\Delta b^{2}}$$

### 2.5. Compounding of Materials and Processing of Parts

In a first study regarding the effect of wood sawdust granulometries, MDPE was dry blended with wood (pre-dried in a Binder oven at 50 °C during 6 h) in the proportions of 90/10 and 70/30 wt%, respectively and processed by rotational molding.

For the processing of the parts, a laboratory rotational molding equipment with characteristics of a Rock and Roll and Shuttle machines was used, with an aluminum mold with parallelepipedic dimensions of 140 mm length, 90 mm width, 90 mm height and 5 mm of mold wall thickness. This is a prototype machine developed at the Department of Polymer Engineering at the University of Minho [48]. In this type of machine, the mold rotates 360° through the axis of the support arm and the oven performs a swinging motion of 45 degrees to the right and to the left perpendicular to the support arm, allowing the spreading of the material for the ends of the part. The rotation speed of the mold and the pendulum swing were 7 rpm and 1.5 rpm, respectively. For each part, 200 g of material was used. The oven was heated by electrical resistances and set to 300 °C.

The internal air temperature (IAT) of the mold was monitored during processing by a thermocouple type k placed inside the mold through the vent conduct. Upon reaching the peak internal air temperature (PIAT) of 200 °C, cooling by air was applied until the temperature inside the mold dropped to 60 °C before demolding the part.

In a second part of the study, the effect of wood content on the composite material was evaluated at specific wood sawdust granulometries, namely, <150, 150–300 and 300–500 µm. Therefore, MDPE was dry blended with different wood sawdust in the proportions of 90/10, 85/15, 80/20, 75/25 and 70/30 wt%. The same conditions referred previously were applied for the processing of parts.

### 2.6. Microscopy Analysis

The wood sawdust and the impacted fractured WPC parts were analyzed by Scanning Electron Microscopy (SEM) using an Ultra-high-resolution Field Emission Gun Scanning Electron Microscope (FEG-SEM), NOVA 200 Nano SEM, FEI Company (Hillsboro, OR, USA). Secondary electron images were performed with an acceleration voltage of 10 kV. Prior to the analyses the samples were covered with a very thin film (20 nm) of Au-Pd (80–20 wt%), in a high-resolution sputter coater, 208HR Cressington Company (Watford, UK), coupled to a MTM-20 Cressington High Resolution Thickness Controller.

To image the part surfaces and defects, an Olympus SZ-PT stereoscopic magnifier (Olympus Corporation, Tokyo, Japan) was used. Surface preparation was not required.

The distribution of wood particles on the MDPE matrix was accessed by bright field microscopy and its influence on the morphology was obtained by polarized light microscopy. Both techniques used a transmission optical microscope Leica DM 2500 P (Wetzlar, Germany), coupled with a Leica Application Suite software (Wetzlar, Germany). Samples were prepared using a Leitz 1401 microtome (Wetzlar, Germany) equipped with a glass slicing knife; 15 μm thick slices were cut along the thickness direction and placed between a microscope glass slide and cover glass. To prevent them from curling up or corrugating, Canada balm was used as a fixing resin.

### 2.7. Mechanical Tests

The mechanical properties of the parts were determined by puncture impact tests, according to the standard ISO 6603-1:2000 and ISO 6603-2:2000 on a CEAST Fractovis Plus instrument falling weight impact tester (INSTRON CEAST, Pianezza, Italy). All the specimens were subjected to the same energy level (147.5 J), with an impact velocity of 4.4 m/s resulting from a drop height of 1 m of a carriage of total mass of 15 kg. The tested specimens were squared with 60 mm side. Eight specimens were tested for each sample.

A statistical analysis was performed using the one-way ANOVA (Analysis of Variance) to determine if differences existed between population means obtained for the same granulometry at different wood contents and for the same wood percentage considering different granulometries. The procedure tested the null hypothesis (Ho) that the average results are equal (suggesting that the incorporation of different wood percentages and different wood granulometries had no effect on the performance) against the alternative hypothesis (Ha) that at least one average result was different. A significance level of 5% was adopted for this study indicating that if the *p*-value of a statistical test was less than 0.05, at least one of the population means was statistically different from the others and the null hypothesis (Ho) was rejected.

### 2.8. Methodology Flow Chart

**Figure 2:**
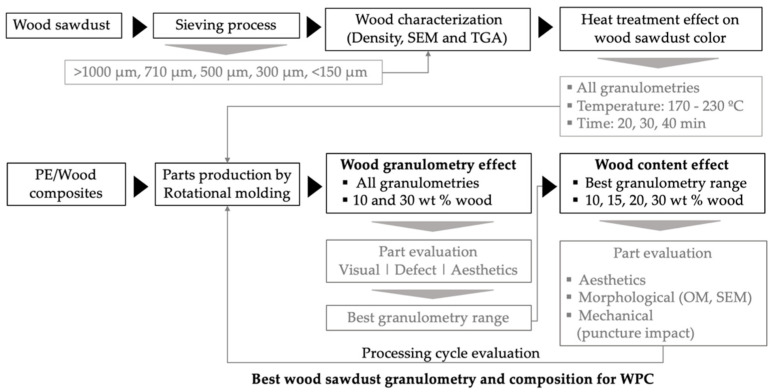
Flow chart summarizing the methodology followed for the work.

Figure 2 represents a flow chart summarizing the methodology adopted for the execution of the experimental work. First, there is the characterization of the wood sawdust raw material upon being sieved in different granulometries; it follows the production of parts by rotational molding and the evaluation of wood granulometry and content on WPC properties. The work flow is set so that the relationship between materials characteristics, processing cycle, structure developed and final properties of wood/PE parts are achieved. The main goal is to identify the characteristics of natural materials that best suits the processing of WPC by rotational molding.

## 3. Results and Discussion

### 3.1. Characterization of Raw Materials

#### 3.1.1. Morphology of Virgin Wood

**Figure 3:**
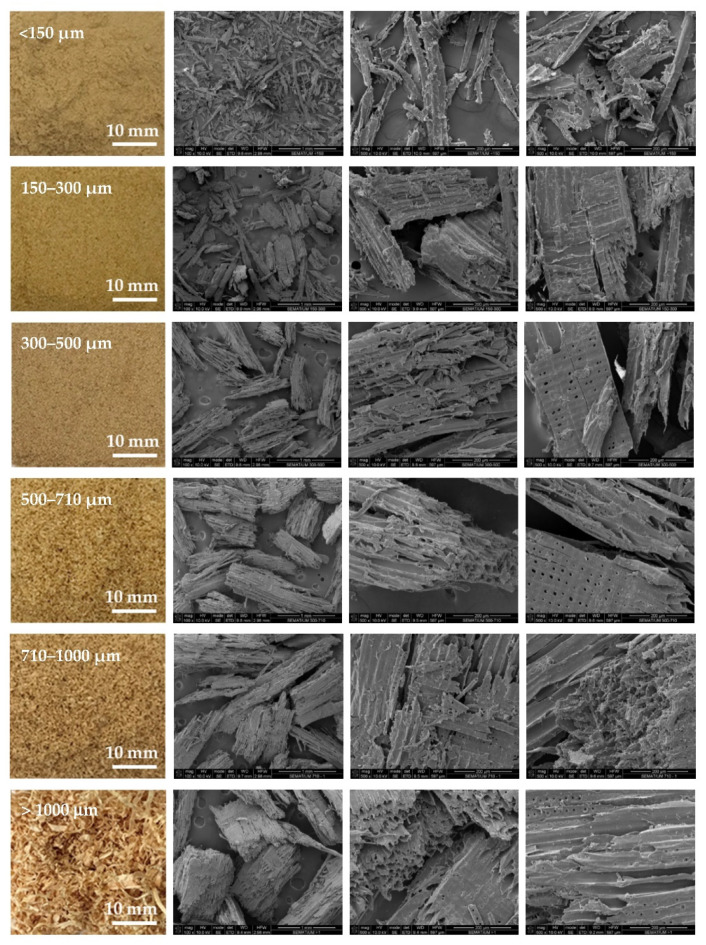
Images of wood sawdust according to its granulometry.

Wood particles were imaged and depicted on Figure 3. The visual appearance of sieved wood (images on the left) resembles a powder for smaller granulometries and small wooden tapes or chips for granulometries greater than 500 μm. SEM images (on the right) shows very irregular wood particles and particles of different shapes and sizes according to their granulometry. The roughness and irregular shape are produced by the cutting and machining processes (sawing or grinding) of wood/timber and are observed in similar way in other published works [32]. Wood granulometries bellow 150 µm (fine particles) are needle-shaped and can be found mostly as individual fibers in its longitudinal structure. With increasing of wood granulometry, particles tend to become a compact brick-shaped structure, with a multicellular structure typical of wood fibers (courser and oversized particles). A transversal lamellar structure is clearly observed in some images. According to Guo et al. [49] biomass materials are characterized by that due to the wood anisotropic structure.

#### 3.1.2. Apparent Density of Wood Sawdust

The apparent density of MDPE and wood sawdust with different granulometries are shown in Table 1. MDPE presents an apparent density of about 0.42 g/cm^3^ which is in the characteristic range of this material [50]. Wood sawdust can vary between 0.22 g/cm^3^ for fine particles size (<150 µm), to 0.18–0.19 for courser particles size (150–1000 µm), and finally 0.08 g/cm^3^ for oversized particles size (>1000 µm). Large-size particles are less likely to pack together as they are ordinarily very bulky, therefore the apparent density is very low. The wood sawdust apparent density is about twice as low as that of MDPE (0.42 g/cm^3^) which may cause difficulties during processing of WPCs by rotational molding.

#### 3.1.3. Thermal Analysis of Wood Sawdust

**Figure 4:**
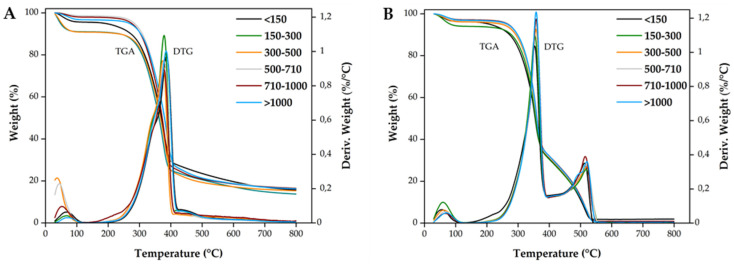
Thermal analysis (TGA) and derivative thermogravimetric analysis (DTG) curves of wood sawdust with different granulometries under: (**A**) nitrogen and (**B**) artificial air atmosphere.

Thermogravimetric analysis (TGA) was used to understand the thermo-degradation behavior of wood sawdust with different granulometries and to identify the temperatures at which the main chemical processes are occurring. TGA was carried under both nitrogen and artificial air atmospheres. The degradation of wood is dominated by the behavior of its three main components: hemicellulose, cellulose and lignin; the proportion of each component in the wood varies depending on the species [51]. The results of the thermogravimetric analysis (TGA) and the respective derivate (DTG) are shown in Figure 4. Relevant data from all materials are summarized in Table 2.

Several regions can be identified in the thermogravimetric curves. Under a nitrogen atmosphere the wood sawdust shows an initial region of mass loss below 240 °C corresponding to the elimination of water (40–100 °C) and a slight decomposition of hemicellulose (150–240 °C). The maximum degradation of hemicellulose takes place at around 310 °C, where a shoulder in the DTG curve appears [52]. Depending on the wood granulometry, the onset of decomposition was observed between 302–313 °C, in this study.

At around 366 °C the main degradation of cellulose occurs and a prominent peak appears at the temperature corresponding to the maximum decomposition rate. This behavior is similar to the literature for other wood species [51,53].

Lignin is relatively more thermal stable when compared to hemicellulose and cellulose, which means this component contribute mainly to the shoulder above 400 °C. Despite that, lignin degrades with increasing temperature, especially between 250 °C and 500 °C [54]. Above 400 °C, cellulose and hemicelluloses are already completely degraded. In the final region, around 500 °C, the rate of mass loss lowers until the wood reduces to ashes. About 16% by weight was retained as residue.

TGA under artificial air atmosphere was also analyzed to understand the behavior of the material in a similar environment to that occurring inside the mold during processing (Figure 4B). After the elimination of water, there is a rapid mass loss between 200 °C and 390 °C. The first peak, between 374 °C and 387 °C corresponds to degradation of polysaccharides composed of cellulose and hemicellulose that have degradation temperatures in the range between 300 °C and 400 °C. The degradation of these two components form a single decomposition step [55]. Lignin is thermally more stable and contributes mainly to the second reaction (512 °C) but oxidation of carbonaceous residues from previous degradation processes also takes through. In the final region, above 520 °C, all wood sawdust reduces to ashes and almost no residue is observed.

The results are suggesting that wood can be processed at temperatures typically used for the processing of polyethylene, between 170–230 °C, with no significant and irreversible changes in the wood chemical composition [52].

#### 3.1.4. Effect of Heat Treatment of Wood Sawdust

**Figure 5:**
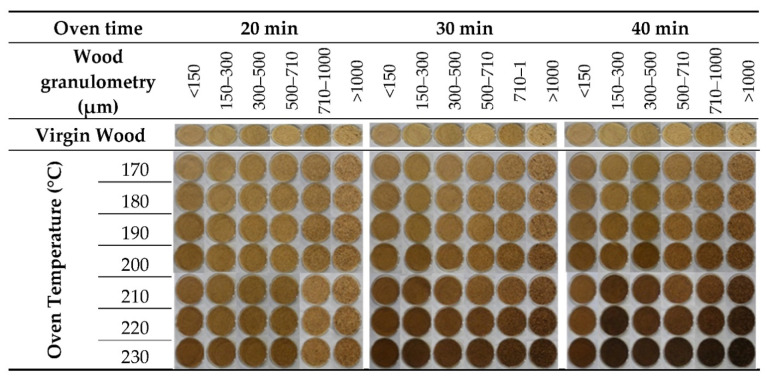
Effect of heat treatment conditions on the color of wood with different granulometries.

**Figure 6:**
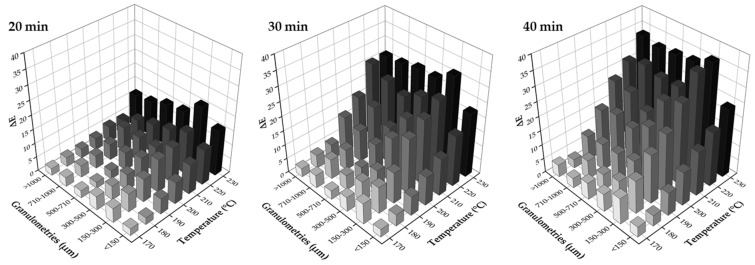
Color variation of wood particles with different granulometries, as a function of temperature and time.

Figure 5 depicts the images of wood sawdust before and after the heat treatment. Wood color presents a light brown color that darkens with increasing temperature and time. The perception of color change is significant for times above 30 min for higher temperatures (above 200 °C). The color variation was evaluated (Figure 6). When increasing the temperature at each time, the following was observed: for <150 µm samples, Δ*E* varied between (2.48–15.97) for 20 min, and (3.64–23.83) for 40 min; for 300–500 µm samples, Δ*E* ranged from (4.83–15.79) for 20 min to (7.17–33.03) for 40 min; and for >1 mm samples Δ*E* ranged between (2.48–11.76) for 20 min and (4.34–33.27) for 40 min. All the values represent a significant color change of wood with heat treatment. However it seem that smaller wood particles are more sensitive to color change at shorter times and less sensitive as the time evolves; the opposite seems to occur for larger size particles. These results show a dependency of wood granulometry on its colour upon heat treatment.

According to Bekhta and Niemz [47], Kučerová et al. [56] and Akkuş and Budakçı [57], the darkening of wood is one of the most visible effects of heat treatment and its intensity depends on the severity of the treatment. This effect is often explained as the result of the formation of colored degradation and oxidation products from hemicelluloses and extractives. It is also stated that heat treatment influences the surface color of different woods and this phenomenon is probably related to the volatilization of color extracts as well as to the oxidation of some chemical constituents of wood, including lignin and polysaccharides. Natural materials are prone to color change as a consequence of initial degradation effect, but not affecting the structural ability of the filler [45,47,56,57].

The characterization of wood sawdust exposed the differences on the size and shapes of wood particles from needle (fine particles) to brick-shaped structures (courser particles). Their apparent density (0.22–0.18 g/cm^3^) is lower than PE (0.42 g/cm^3^) and even more for oversized wood particles (0.08 g/cm^3^). The color of wood changes when exposed to heat treatment at high temperatures and times. These characteristics may result in difficulties on the sintering of WPC during rotational molding and the formation of parts with wood color degradation (darkening) as a result of oxidation products from hemicelluloses and extractives, occurring between 150 to 240 °C, that is within the range of processing window of PE.

### 3.2. Production of Parts by Rotational Molding

#### 3.2.1. Evaluation of Processing Cycle

**Figure 7:**
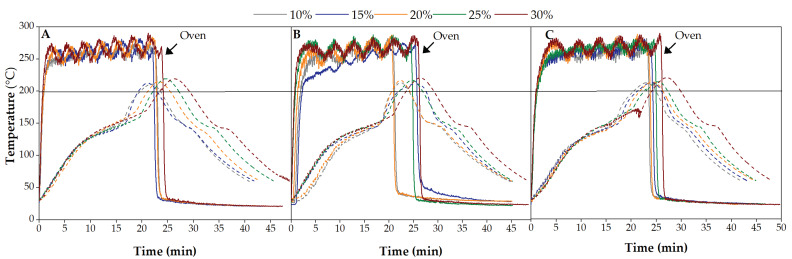
Oven and mold internal air temperature profiles during the processing cycle of MDPE/wood composites by rotational molding. Effect of wood content and its granulometry: (**A**) <150 µm; (**B**) 150–300 µm and (**C**) 300–500 µm.

Figure 7 presents the processing cycle curve of rotomolded parts, by depicting the oven temperature and the internal air temperature of the mold (IAT) as a function of time. In all cases, although the setup oven temperature was defined for 300 °C, the maximum oven temperature reached was 287 °C. The internal air temperature of the mold shows a typical curve, with a rapid increase in the temperature followed by a plateau observed between 120 and 160 °C, which represents the moment of melting and adhesion of the PE/wood to the mold surface. The presence of natural fibers causes a delaying effect on plastic particle coalesce during the sintering process, as observed in the plot [58]. When the cooking stage is completed and PIAT is reached (setup at 200 °C) the mold is transferred to the cooling chamber; the temperature drops and a small plateau occurs due to the crystallization of PE. It follows the solidification of the material until the part is demolded.

During processing cycle an overshooting of PIAT is observed that is related to the presence of wood on the composite (a line is depicted at setup PIAT, 200 °C). The heat transferred from the mold to the particles is delayed and becomes trapped inside the mold during cooling, leading to an overshooting of PIAT and longer cycle times. The higher the wood content on the composite, the greater is its effect on the cycle time. Moreover, a color change on the part is observed, as the material is inside the mold for about 20 min at temperatures between 170 °C and 225 °C (real PIAT). The color of the part is darker for granulometries <150 and 150–300 μm than for 300–500 μm and above (Table 3), following the same trend observed on the heat treatment studies. Related results were observed in previous studies in PE/cork composites [45]. 

#### 3.2.2. Effect of Wood Sawdust Granulometry

Table 3 depicts the appearance of rotomolded parts produced with different wood granulometries and compositions (10 and 30 wt%). Parts of good quality were obtained; different colors and textures were achieved depending on the granulometry used. Parts produced with fine particles were darker in color, following the same tendency observed in the heat treatment studies. Moreover, when increasing the wood content to 30 wt%, parts were no longer homogeneous in its color and a color texture appeared.

The increase in wood granulometry resulted in the reduction of the sintering capability of the composite increasing its porosity, wall thickness and surface defects. Some defects are illustrated in Table 4 for reference. Parts processed with 500–710 µm and 710–1000 µm have a plastic shiny look, plastic touch and milky surface in some regions, resulting from the presence of PE on the outer layer. Furthermore, the increase in wood content resulted in the appearance of defects such as pinholes, heterogeneous surface, surface roughness and visible wood particles.

These results are in good agreement with a very recent work of Arribasplata-Seguin et al. [32] that have studied in great detail the sintering process of parts by rotational molding when varying the processing conditions applied (oven temperature and time) and the wood particles size of untreated capirona and pine wood. They showed that irregular surfaces can be obtained by the lack of sintering time, low temperatures or large wood granulometries. Therefore, the sintering process develops more efficiently as wood particles size decreases or the oven temperature and heating time increases.

Torres and Aguirre [31] also showed a tendency for the natural fibers to remain in the inner surface of the molded part, as observed in Table 4, image (C) and (E). To solve this problem, it is suggested to add a second layer of unreinforced polymer that covers and sinters on the inner surface to produce an inner smooth surface.

Particles larger than 1 mm were excluded from the study, since they presented a non-homogeneous color, numerous voids, lack of wood/polymer wetting and adhesion, and loose wood material inside the part. These results may be explained by the great difference in the apparent density of the materials, which translates into very poor sintering and the appearance of defective parts.

It is important to notice that color of parts is greatly dependent on the processing cycle of each part; thus, it is expectable to observe some color variation on the reproduction of parts. The parts with better appearance, look and touch, that revealed some similarity to wood sawdust were those with granulometries of <150 μm, 150–300 μm and 300–500 μm. Therefore, the studies that follows were carried only with these wood granulometries.

#### 3.2.3. Effect of Wood Content on Part Quality

The wood content has a major effect on the part’s final appearance. As observed in Table 5, there is a change from homogeneous brown color at low wood weight percentages (10 and 15%) to a color texture above 20%.

The major reason for that seems to be the inability of polymer to create a uniform and smoother layer at the outer surface by the presence of wood particles (Table 6). The adhesion between plastic particles begin much earlier in WPC that contain lower quantities of wood (up to 15%). For those, a smooth external surface with roughness identical to PE is obtained. Surface defects are more abundant at high wood contents, such as pinholes and surface roughness. Therefore, they change the way light is reflected on the sample surface, giving the sensation of a color change, tint or shade. The internal surface also becomes rough with increase of wood content and its granulometry, since no enough polymer is available to wet all the wood particles (Table 6) [32]. The inability of the WPC to compact properly, as rotational molding process is unable to provide that due to absence of any shear stresses during the processing cycle [32], causes the thickness of the part to increase significantly (results summarized in Table 5). Rotational molding is prone to have voids along the thickness direction due to air trapped during cooling, that could be enhanced with wood natural moisture [27]. However, according to Ward-Perron et al. most of the humidity evaporates before the polymer starts melting and sticks to the mold walls, resulting in its elimination before the end of rotational molding heating cycle [59]. Hence, the porosity observed is the result of high wood contents and lack of good sintering of materials, as plastic particles are unable to wet wood properly [32].

The production of WPC parts reveals the importance of wood granulometry and wood content on the part quality. WPC processed well at temperatures used for PE, however the large cycle time applied at high temperatures caused some wood color darkening. Wood granulometries bellow 500 µm resulted in parts of good quality, brown in color and with good aesthetic appeal; 500 to 1000 µm resulted in defective parts, namely, plastic touch and shiny look, inhomogeneous surfaces and visible wood particles. The wood content played an important role on color; up to 15% a homogeneous brown color was obtained and above 20% color texture appeared. Surface defects were more abundant at high wood contents, such as pinholes and surface roughness, due to WPC sintering difficulties.

### 3.3. Characterization of Rotomolded Parts

#### 3.3.1. Morphology of Parts

**Figure 8:**
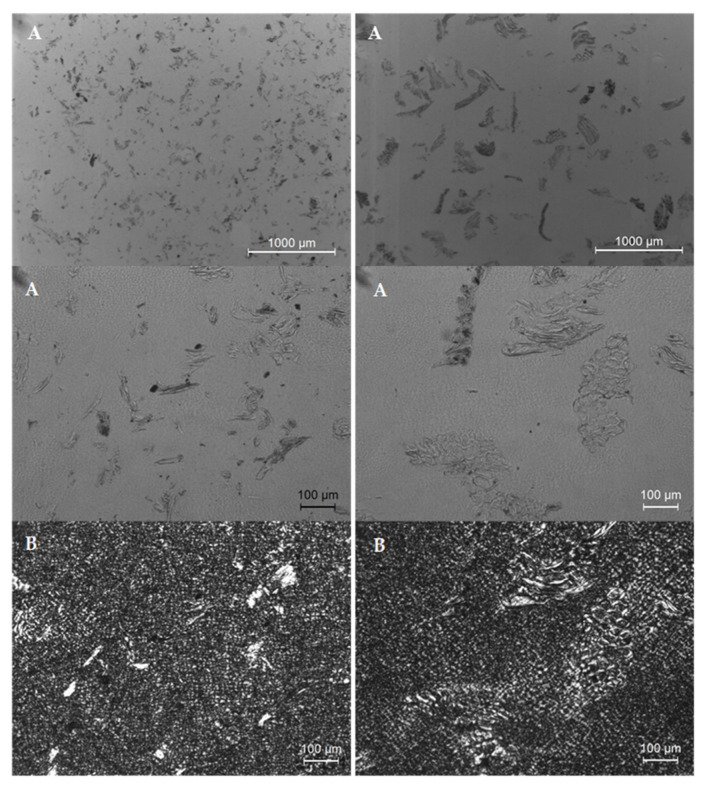
Optical microscopy images of MDPE/10 wt% wood with granulometries of <150 μm (images on the **left**) and 300–500 µm (images on the **right**), obtained by (**A**) bright field microscopy and (**B**) polarized light microscopy.

Optical microscopy images of PE/10 wt% wood parts with 150 and 300–500 µm are depicted in Figure 8. Difficulties on cutting samples containing larger wood contents made unfeasible to illustrate them. Wood particles are well distributed on the polymer matrix, being clear the difference between wood granulometries on each case. No agglomerates are observed. Heterogeneities on the wood sizes and shapes are expected, from the characteristics of the virgin wood material. The morphology of PE is characterized by a very small and uniform spherulitic structure, which seems to indicate that wood has no effect on the crystallization behavior of PE. Some voids are observed, that are common in rotational molding products [27].

**Figure 9:**
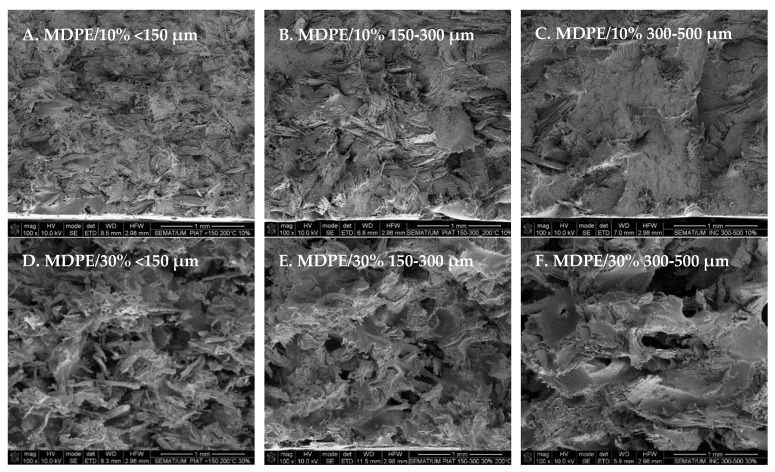
Effect of wood granulometry and wood content on the morphology of MDPE/wood composites.

**Figure 10:**
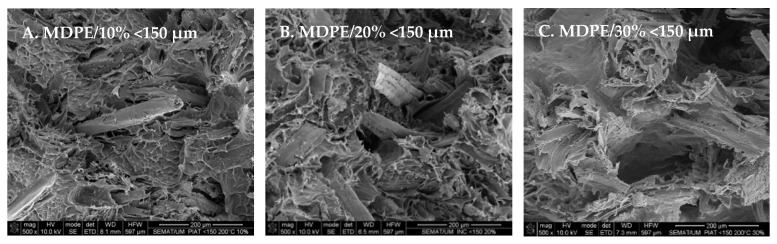
Effect of wood content on the morphology of MDPE/wood composites, with wood granulometry of <150 µm.

**Figure 11:**
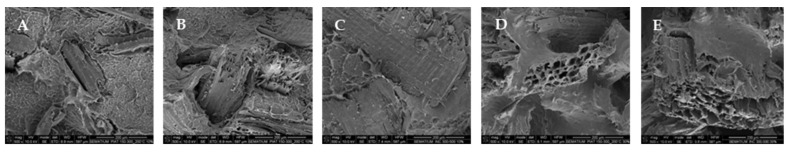
Morphological details of MDPE/wood composites. From **left** to the **right**: (**A**) fiber mechanically anchored to the matrix (MDPE/10% 150–300 μm); (**A**,**B**) lack of interfacial adhesion of wood to polymer (MDPE/10% 150–300 μm); (**C**) fiber on its transversal direction embodied on the polymer (MDPE/10% 300–500 μm); (**D**) wood bundle well embodied on the polymer surrounded by large voids (MDPE/30% 150–300 μm); (**E**) wood bundle poorly wet by the polymer (MDPE/30% 300–500 μm).

SEM images (Figure 9) show wood particles well distributed on the matrix, regardless of the wood granulometry used. Fibers are very abundant in the case of wood granulometry <150 µm given their size compared to the other two cases, where it is easily seen the PE matrix and its ductile facture (Figure 9 and Figure 10). The increase of wood content causes the increase of porosity on the matrix; the appearance of single fibers or small fiber bundles pullout from the matrix and the observation of wood bundles that are poorly wet by the polymer. These results illustrate well the difficulties on sintering the PE/wood parts when high wood contents are used (Figure 9D–F and Figure 11D,E). Despite the weak interfacial strength between the wood and the matrix, the irregular shape of wood bundles or brick-shape wood, results in the mechanical anchoring of the wood to the matrix, as observed in Figure 11.

#### 3.3.2. Mechanical Properties of Parts

**Figure 12:**
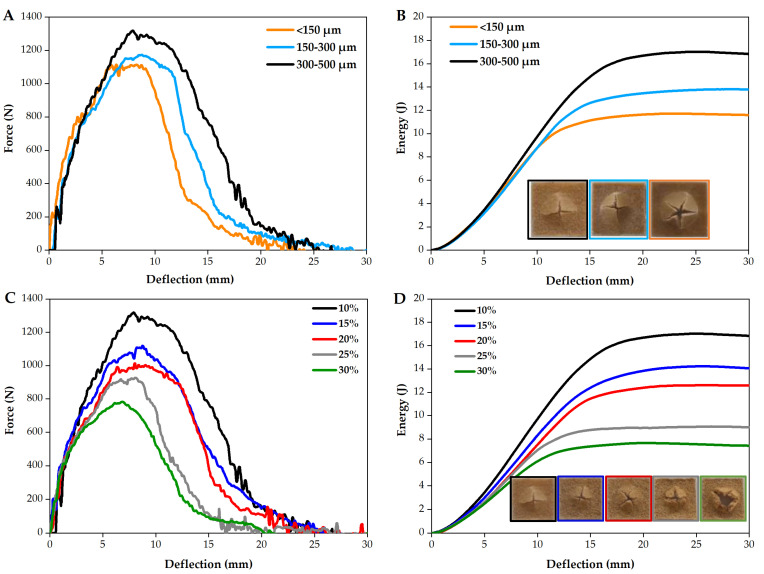
Force-deflection curve and energy-deflection curve of PE/wood as a function of wood granulometry (**A**,**B**) for parts with 10% wood and (**C**,**D**) wood content for parts processed with 300–500 µm wood.

Figure 12 depicts the mechanical impact behavior of the composite materials as a function of wood granulometry (Figure 12A,B) and the percentage of wood content (Figure 12C,D). The force and the energy to maximum force as a function deflection are shown together with the fracture surfaces of the parts. All parts suffer a failure by yielding followed by crack growth.

**Figure 13:**
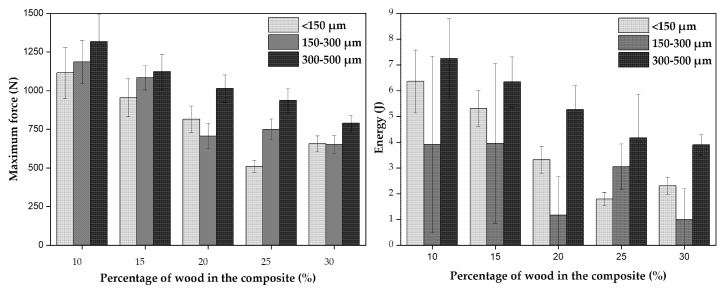
Effect of wood content and granulometry on the mechanical impact properties of MDPE/wood parts, namely, maximum force and energy to maximum force.

These results are analyzed in Figure 13. The increase of wood granulometry, increases the force needed to yield to a fracture, whereas the increase of wood content decreases it. The same tendencies are observed for the energy at maximum force. The results may present a large standard deviation indicating the heterogeneity of the composites obtained upon processing.

ANOVA results are presented in Table 7 and Table 8, respectively for the force and energy. The nule hypotheses is rejected in most of the conditions which shows that the population means studied are statistically different. With the exception on two cases, namely, the force for parts with 10% wood and the energy for parts with 15% wood, as they have *p*-value > 0.05.

According to the morphological studies, larger particles with irregular brick-shape, have better mechanical anchoring to the polymeric matrix, leading to improved mechanical properties as compared to small particles. These results are in good agreement with Dikone and Luyt [60] and Hanana et al. [37]. Regarding the effect of wood content, it is clear that low wood contents have a positive effect on the composite impact behavior. The impact force required to fracture a PE/300–500 µm wood sample reduces by 40% from 10 wt% (1316 N) to 30 wt% (789 N). This tendency is related to the lack of wettability and adhesion between materials as observed previously and also by other authors [32]. The presence of voids and porous structure due to poor sintering of material contributes to stress concentration points and a weak ability to transfer energy between them, causing the failure of the composites and composites with lower mechanical properties [37,61].

In conclusion, parts with 10% of wood particles, regardless of their size (according to ANOVA results) seem to be the best combination to get the higher mechanical properties in MDPE/wood composites, processed by rotational molding. For mechanical impact forces up to 1000 N, it is possible to consider composites containing up to 20 wt% of wood in its composition, but in this case, aesthetics of the part may be affected as reported earlier. These results are in good agreement with Cisneros-Lopez et al. [33] and Hanana et al. [44]. The study also shows that particle granulometries of 300–500 µm are those presenting the best properties among all particle’s sizes studied.

## 4. Conclusions

The polyethylene wood sawdust composites produced by rotational molding were investigated. The effect of wood granulometry (<150, 150–300, 300–500, 500–710, 710–1000, >1000 µm) and wood composition (10–20–30 wt%), on the processing and properties of PE/wood composites were analyzed. Parts of good quality with a natural wood appearance, look and touch, were obtained for granulometries bellow 500 μm. Higher granulometries resulted in part defects such as pinholes, heterogeneous surfaces, roughness and unwetted fibers, and therefore are not recommended. Wood content reduced the impact properties by 40% from 1316 N (10 wt%) to 789 N (30 wt%). This behavior is related to the poor sintering of materials revealed by a porous structure, lack of wettability and poor adhesion between PE/wood. The inability of WPC to sinter properly, causes the thickness of the part to increase. Among all, particles of 300–500 μm were those presenting better mechanical performance as good anchoring of irregular brick-shaped benefited those from a morphological point of view. The color of the parts was affected by the processing cycle applied. In conclusion, WPC are suitable materials for rotational molding applications and the production of sustainable products with differentiated wood aesthetics and color varying with the thermal processing cycle. The characteristics of the parts are affects by the granulometry and content of sawdust.

## Figures and Tables

**Table 1:** Apparent density of MDPE and wood with different granulometries. Images correspond to 10 g of each material.

	MDPE	Wood Sawdust					
Granulometry (µm)	<500	<150	150–300	300–500	500–710	710–1000	>1000
Apparent Density (g/cm^3^)	0.42	0.22	0.19	0.19	0.18	0.18	0.08
Powders	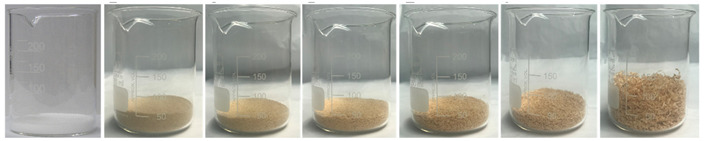						

**Table 2:** Summary of TGA data for each wood granulometry.

Granulometry (µm)	Onset Temperature of Decomposition (°C)		Temperature at Maximum Decomposition Rate (°C)		Residue at 800 °C (%)	
	Nitrogen	Air	Nitrogen	Air	Nitrogen	Air
<150	302.0	287.2	366.3	379.8	15.7	1.9
150–300	317.2	295.7	366.1	373.5	13.7	0.6
300–500	312.7	301.8	367.7	379.5	15.3	0.6
500–710	311.5	305.7	368.5	385.7	16.6	0.5
710–1000	313.5	308.3	368.2	379.2	16.4	0.9
>1000	313.4	312.0	365.4	387.3	16.2	0.4

**Table 3:** Images of parts produced by MDPE/wood composites using 10 and 30% of each wood granulometry.

Granulometry	<150 µm	150–300 µm	300–500 µm	500–710 µm	710–1000 µm
Whole parts 10%	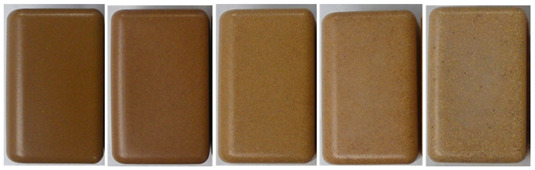				
Whole parts 30%	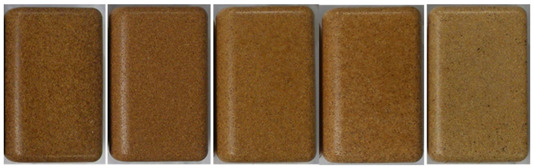				

**Table 4:** Main defects of parts produced with MDPE/wood composites with large wood granulometries: (A) MDPE/10% 500–710 µm; (B) MDPE/30% 500–710 µm; (C) MDPE/30% 710–1000 µm; (D) and (E) MDPE/10% >1 mm.

A. Plastic Shiny Look	B. Pinholes and Milky Surface	C. Porous Inner Surface	D. Surface Roughness and Porosity	E. Visible Wood Particles
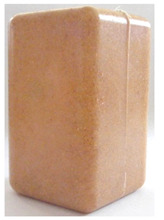	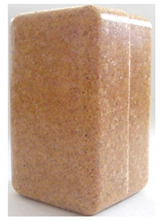	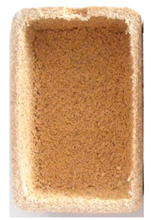	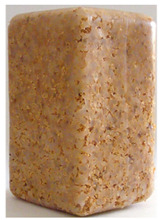	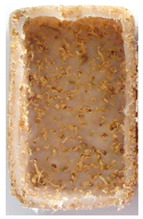

**Table 5:** Images of parts produced by MDPE/wood composites with 10, 15, 20, 25 and 30% wood content, for <150 µm, 150–300 µm and 300–500 µm wood granulometries.

	Wood Content						
	10%	10%	15%	20%	25%	30%	30%
<150 µm	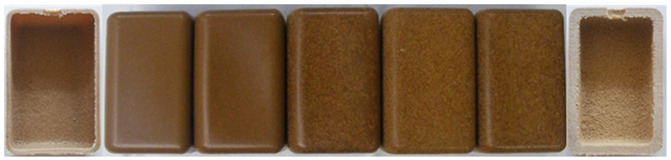						
Thickness (mm)		4.4 ± 0.4	4.7 ± 0.6	5.4 ± 0.6	6.8 ± 1.0	7.2 ± 1.4	
150–300 µm	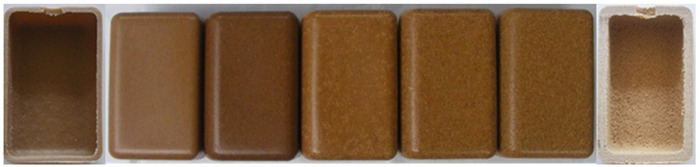						
Thickness (mm)		4.5 ± 0.5	4.9 ± 0.6	5.1 ± 0.7	5.9 ± 0.5	7.3 ± 0.6	
300–500 µm	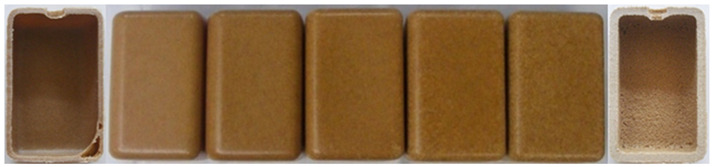						
Thickness (mm)		4.2 ± 0.2	4.7 ± 0.6	5.5 ± 0.6	6.6 ± 0.6	8.3 ± 1.1	

**Table 6:** Effect of wood granulometry and wood content on the surface characteristics of PE/wood parts.

		Wood Content			
		10%	15%	20%	30%
<150 µm	Outer Surface	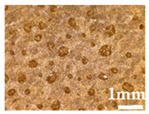	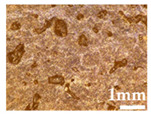	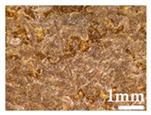	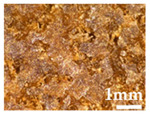
	Inner surface	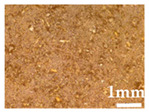	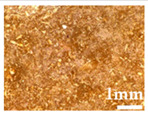	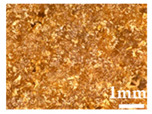	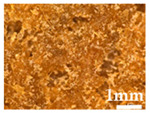
150–300 µm	Outer Surface	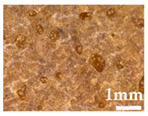	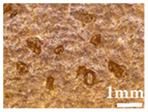	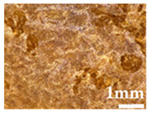	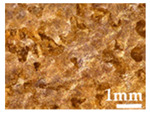
	Inner surface	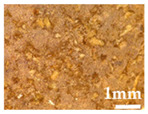	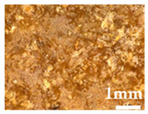	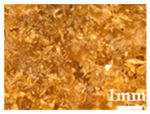	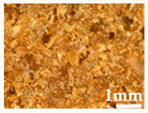
300–500 µm	Outer Surface	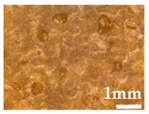	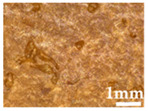	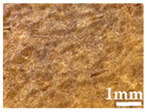	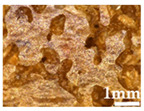
	Inner surface	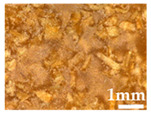	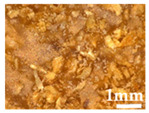	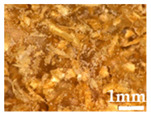	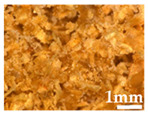

**Table 7:** One-way ANOVA for comparison of the population means regarding the maximum force.

Mean Maximum Force (N)							
	10%	15%	20%	25%	30%	F Value	*p*-Value
<150 µm	1115.4	9547.3	815.2	509.9	656.6	37.5	3 × 10^−12^
150–300 µm	1186.4	1083.2	705.5	749.3	652.2	51.0	4 × 10^−14^
300–500 µm	1317.0	1122.3	1013.2	936.6	789.0	23.1	2 × 10^−9^
F value	2.9	4.8	23.4	81.0	14.9		
*p*-value	0.080	0.019	4 × 10^−6^	1 × 10^−10^	9 × 10^−5^		

**Table 8:** One-way ANOVA for comparison of the population means regarding the energy at maximum force.

Mean Energy (J)							
	10%	15%	20%	25%	30%	F Value	*p*-Value
<150 µm	6.4	5.3	3.3	1.8	2.3	55.3	1 × 10^−14^
150–300 µm	3.9	3.9	1.2	3.1	1.0	2.8	0.039
300–500 µm	7.2	6,3	5.3	4.2	3.9	10.1	1 × 10^−5^
F value	4.0	2.7	25.8	8.0	14.9		
*p*-value	0.034	0.089	2 × 10^−6^	0.003	9 × 10^−5^		

## Data Availability

Not applicable.
